# Effect of climate dataset selection on simulations of terrestrial GPP: Highest uncertainty for tropical regions

**DOI:** 10.1371/journal.pone.0199383

**Published:** 2018-06-21

**Authors:** Zhendong Wu, Niklas Boke-Olén, Rasmus Fensholt, Jonas Ardö, Lars Eklundh, Veiko Lehsten

**Affiliations:** 1 Department of Physical Geography and Ecosystem Science, Lund University, Lund, Sweden; 2 Department of Geosciences and Natural Resource Management, University of Copenhagen, Copenhagen, Denmark; 3 Swiss Federal Institute for Forest, Snow and Landscape research (WSL), Birmensdorf, Switzerland; Universidade de Aveiro, PORTUGAL

## Abstract

Biogeochemical models use meteorological forcing data derived with different approaches (e.g. based on interpolation or reanalysis of observation data or a hybrid hereof) to simulate ecosystem processes such as gross primary productivity (GPP). This study assesses the impact of different widely used climate datasets on simulated gross primary productivity and evaluates the suitability of them for reproducing the global and regional carbon cycle as mapped from independent GPP data. We simulate GPP with the biogeochemical model LPJ-GUESS using six historical climate datasets (CRU, CRUNCEP, ECMWF, NCEP, PRINCETON, and WFDEI). The simulated GPP is evaluated using an observation-based GPP product derived from eddy covariance measurements in combination with remotely sensed data. Our results show that all datasets tested produce relatively similar GPP simulations at a global scale, corresponding fairly well to the observation-based data with a difference between simulations and observations ranging from -50 to 60 g m^-2^ yr^-1^. However, all simulations also show a strong underestimation of GPP (ranging from -533 to -870 g m^-2^ yr^-1^) and low temporal agreement (r < 0.4) with observations over tropical areas. As the shortwave radiation for tropical areas was found to have the highest uncertainty in the analyzed historical climate datasets, we test whether simulation results could be improved by a correction of the tested shortwave radiation for tropical areas using a new radiation product from the International Satellite Cloud Climatology Project (ISCCP). A large improvement (up to 48%) in simulated GPP magnitude was observed with bias corrected shortwave radiation, as well as an increase in spatio-temporal agreement between the simulated GPP and observation-based GPP. This study conducts a spatial inter-comparison and quantification of the performances of climate datasets and can thereby facilitate the selection of climate forcing data over any given study area for modelling purposes.

## Introduction

Biogeochemical models are widely used to refine and upscale field measurement of spatiotemporal carbon exchange and great advances have been made in developing these models in the last decade (e.g. [1–4]). Furthermore, biogeochemical models are used to predict future carbon budgets under different scenarios providing descriptions of future potential biogeochemical conditions essential to assess socioeconomic, technological and environmental conditions, emissions of greenhouse gases and aerosols, as well as climate [5, 6]. These models are usually driven by climate data and simulate the spatio-temporal vegetation dynamics as well as the carbon fluxes and water flows through the ecosystem [7–9]. However, the choice of historical climate dataset input can cause considerable uncertainty in estimated Gross Primary Production (GPP, the total amount of carbon captured by vegetation via photosynthesis) with outputs fluctuating by 9% to 20% [10–12]. The choice of the climate dataset also has a pronounced impact on the spatial patterns of simulated GPP [11, 13]. Therefore, the selection of historical climate datasets plays a crucial role in both exploring and quantifying the ecosystem response to climate through ecosystem models.

Uncertainty among different historical climate datasets exist at present, which mainly differ in the source and the processing of the raw data. Such climate grids are derived either from quasi-point based measurements and subsequent spatial interpolation, model-based reanalysis, or generated as an observational-reanalysis hybrid. Measurement-based datasets, e.g. Climatic Research Unit (CRU; [14]), are produced by statistical interpolation of climate station records, e.g. by using the Climate Anomaly Method [15]. Reanalysis is a different approach that uses a combination of meteorological forecast model output and assimilated observations. Unlike the observational based datasets, which are based on statistical principles, reanalysis datasets are built on physical principles describing the variable in question [16], by combining climate model output with a large amount of different observational data, such as land cover, trace gases, aerosols, solar variations and wind speed. As a third type, observational-reanalysis hybrid datasets combine observations and reanalysis data [17, 18].

This study is motivated by two factors: Firstly, as there is no general agreement about which historical climate data set is most suitable for driving biogeochemical models, several of the currently available historical climate datasets are widely used for contemporary research on estimations of GPP [2, 4, 19–21], yet very few studies (e.g. [12,13]) have investigated the difference in reproducing the carbon cycle associated with the use of climate datasets. The suitability of contemporary historical climate datasets for accurately estimating GPP at global and regional scales is therefore currently not well known. Secondly, users of biogeochemical models normally rely on the climate dataset for which the model was calibrated to reproduce the carbon cycle with the least uncertainty for a particular region. However, climate datasets might vary in quality in a spatially explicit way governed by the processing algorithm and underlying density of available calibration points. There are also incidences in which the user can not choose the dataset for which the model was calibrated, e.g. in a model comparison study where different models need to be driven by similar input data, or if the calibration dataset has a lower temporal resolution than what is required by a specific task. Therefore, this study fills a current research gap by evaluating the six most commonly used climate datasets (CRU, CRUNCEP, ECMWF, NCEP, PRINCETON, WFDEI; See Methods) and their relative performance of estimating terrestrial GPP within a spatially explicit biogeochemical model to highlight the associated uncertainty. Such quantification is expected to facilitate the selection of relevant climate forcing data when performing GPP modelling over any given study area.

Here we focus on terrestrial GPP, a fundamental driver of plant biochemical processes and an important component of the global carbon cycle [22]. In biogeochemical models, GPP represents the origin of carbon within the system, which controls many other processes (e.g. carbon allocation, plant allometry and tissue turnover) in the models [2, 4, 19–21]. GPP is mainly influenced by climate forcing (e.g. temperature, water, light, and atmospheric CO_2_ concentration), and also influenced by nutrient availability and disturbances (e.g., storms, harvesting, and insect attacks). We use the biogeochemical model Lund-Potsdam-Jena General Ecosystem Simulator (LPJ-GUESS [3, 21]) as a representative model to simulate GPP and compare results to an independent observation-based GPP product, (even though climate data is also used to generate the observation-based GPP product; see discussion for more details). The observation based GPP product is derived from a global network of eddy covariance measurements in combination with remote sensing data and is used as a benchmark to evaluate the performances of the climate datasets. We analyze the differences in magnitude and spatio-temporal pattern of GPP globally and over five vegetated land cover classes to assess their relative performance of reproducing the carbon cycle during the period 1982–2010.

## Methods

### Biogeochemical model (LPJ-GUESS)

LPJ-GUESS is a process-based biogeochemical model, designed for both regional and global studies [21]. It requires time series data of climate forcing (i.e. air temperature, precipitation and shortwave radiation) and atmospheric carbon dioxide concentrations as input. It explicitly represents vegetation cover (by indicating the occurrence of Plant Functional Types, PFTs), age cohorts, gap dynamics and biogeochemical cycles. Vegetation physiological processes such as photosynthesis, canopy conductance, phenology, and carbon allocation are incorporated in the model. LPJ-GUESS uses a detailed individual-based representation of forest stand structure and dynamics for PFTs co-occurring in a number of patches or local stands, representative for the landscape of a grid cell. Each PFT is characterized by properties such as growth form, leaf phenology, life history and bioclimatic limits, which govern their performance and competitive interactions under the forcing conditions and realized ecosystem state of a particular grid cell [20, 23]. In total 11 PFTs are used within this study and their prescribed parameters can be found in Smith *et al*. [3]. We employ LPJ-GUESS version 3.0 [3] which uses nitrogen dynamic based on the CENTURY model [24, 25]. All simulations are initialized with a 500 years spin-up, which comprises an internal 40000 years spin-up mechanism for soils, to equilibrate soil and vegetation pools, by recycling de-trended 1979–2010 climate forcing fields and applying constant CO_2_ concentration and nitrogen deposition from the first year (1979). Subsequently transient GPP is simulated with time evolving CO_2_ concentrations from Keeling and Whorf [26], nitrogen deposition from Lamarque *et al*. [27] and climate forcing. The managed land use fraction is obtained from Hurtt *et al*. [28].

### Historical climate datasets

We force LPJ-GUESS with six different historical climate datasets (Table 1) to simulate global terrestrial GPP. The datasets differ in their spatial and temporal resolution, available time period, and how they are derived. They are derived from quasi-point based measurements (CRU and CRUNCEP), model-based reanalysis (NCEP and ECMWF), or hybrid datasets combining both observation and reanalysis data (WFDEI and PRINCETON). To enable a direct comparison between simulations, the datasets are rescaled to a common spatial (0.5 decimal degree) and temporal scale (monthly observations, since CRU is provided only as monthly data). To allow this, we use bilinear interpolation to convert NCEP to 0.5 degrees, and temporally convert CRUNCEP, ECMWF, NCEP, PRINCTON and WFDEI from daily to monthly time scales. These monthly datasets are subsequently interpolated to daily values uniformly within LPJ-GUESS [21]. Since LPJ-GUESS treats all dataset in the same way, it offsets at least part of interpolation induced bias. We use the common time period 1979–2010 and the climate variables precipitation, shortwave radiation, and air temperature for all datasets. For CRU, the cloud cover is converted to shortwave radiation within the biogeochemical model using the method by Harris *et al*. [29]. All data are available on the DataGURU server (https://dataguru.lu.se/).

**Table 1:** Main datasets used, type, spatial resolution and time period.

Dataset	Type	Spatial resolution	Time period		Reference
CRU TS 3.21	Climate	0.5 degree	1901–2012		[14]
CRUNCEP v5	Climate	0.5 degree	1901–2013		[30]
ECMWF/ERA Interim	Climate	0.5 degree	1979–2014		[31]
NCEP-DOE II	Climate	2.5 degree	1979–2014		[32]
Princeton_V2	Climate	0.5 degree	1901–2012		[17]
WFDEI_GPCC	Climate	0.5 degree	1979–2010		[18]
ISCCP	Radiation	0.5 degree	1984–2000		[33]
JUNG11	GPP	0.5 degree	1982–2011		[34]

### Observation-based GPP product

We evaluate the simulated GPP with a benchmark GPP product derived from eddy covariance measurements from Jung *et al*. [34] (herein after, JUNG11). JUNG11 is derived from long-term and high-quality measurements of carbon dioxide, water, and energy fluxes from the Flux Network (FLUXNET). These *in situ* measurements are very sparse at the global scale, and need to be extrapolated in space, in order to be applicable for global scale studies. Jung *et al*. [34] used a semi-empirical model (Model Tree Ensembles; MTE), to upscale measurements from local to global scales using remotely sensed fraction of Absorbed Photosynthetically Active Radiation (fAPAR), gridded climate, and the Synergetic land cover product (SYNMAP). The long-term mean climatic information used in JUNG11 is derived from CRU data [35] as well as other climate datasets, e.g. global grids of monthly precipitation from GPCC [36] and the ECMWF ERA interim reanalysis product of Simmons *et al*. [37]. In this study, the observation-based JUNG11 dataset is assumed to represent “true” information of GPP, though we are well aware of the uncertainties related to this product, e.g. the uncertainties originating from flux measurements and upscaling station-based fluxes to global scale [34].

### Comparison of GPP estimates with the different climate datasets

Two of the most commonly used metrics to compare model estimates with observations, are the Pearson correlation coefficient (r) and root mean square deviation (RMSD). Despite their popularity, both metrics have disadvantages, as r only measures the strength of relationship between two data series, but does not indicate if the data series have similar magnitude. RMSD, on the other hand, assesses if the absolute values of two series match, but does not indicate the agreement of pattern of the data series. Moreover, RMSD is dimensional, which hampers inter-comparability between analysis outputs. To consider both the strength of the relationship and similarity in magnitude, Willmott [38] proposed an index of agreement (IoA, *d*) for evaluating model prediction (*P*) against measured observations (*O*), as follows:

$$d=1-\frac{\sum_{i=1}^{n}{\left(P_{i}-O_{i}\right)}^{2}}{\sum_{i=1}^{n}{\left({|P}_{i}-\bar{O}|+|O_{i}-\bar{O}|\right)}^{2}}$$ (1)

The upper limit of IoA (*d)* is one which indicates a perfect match, while the lower limit is zero which indicates complete disagreement. The metric describes the relative co-variability of *P* and *O* related to the observed mean ($\bar{O}$).

We use Willmott’s IoA to quantify the match between simulated GPP and JUNG11. If two datasets differ by only 5% we define their result as equal to allow for some small statistical differences. Furthermore, we use the correlation coefficient (r) and annual means as an evaluation measure to assess the IoA result and link it to temporal patterns or magnitude differences. The comparisons are conducted globally and for five vegetated land cover classes derived from Ahlström *et al*. [39] during 1982–2010 (S1 Fig).

### Radiation correction

Simulated GPP has its largest deviation among the datasets for the tropical region (according to initial calculations; S2 Fig). A previous study [12] also revealed that climate dataset induced uncertainty in GPP estimates simulated by LPJ-GUESS was mainly caused by the uncertainty in shortwave radiation over tropical regions. Therefore, we test whether bias correcting the shortwave radiation variable of the tested climate datasets using the International Satellite Cloud Climatology Project (ISCCP) radiation data results in an improvement of the simulated GPP. The ISCCP radiation product is derived from an advanced radiative transfer model (NASA Goddard Institute for Space Studies) by using improved cloud climatology and ancillary data sets [33, 40]. ISCCP has been used as the reference radiation in previous studies, e.g. [41, 42].

The bias correction of shortwave radiation is done only for tropical regions, where simulated GPP is particularly sensitive to shortwave radiation [12], the largest deviation in simulated GPP is shown (S2 Fig) and the largest difference in shortwave radiation among the datasets is present. The differences of the monthly mean shortwave radiation between the tested climate datasets and the ISCCP radiation during the common 17 years (1984–2000) is used to correct the original monthly data from the tested climate datasets ($R_{t}^{orig}$) during 1982–2010 using Eq 2.

$$R_{t}^{corr}=R_{t}^{orig}+(\bar{R^{ref}}-\bar{R^{orig}}),$$ (2)

where $R_{t}^{corr}$ is the bias corrected shortwave radiation for month *t*. $\bar{R^{orig}}$ and $\bar{R^{ref}}$ are the monthly mean of the tested climate datasets and the ISCCP radiation during 1984–2000, respectively. This bias correction adjusts for biases in annual averages and seasonal distribution, while preserving the inter-annual variability.

## Results

**Fig 1:**
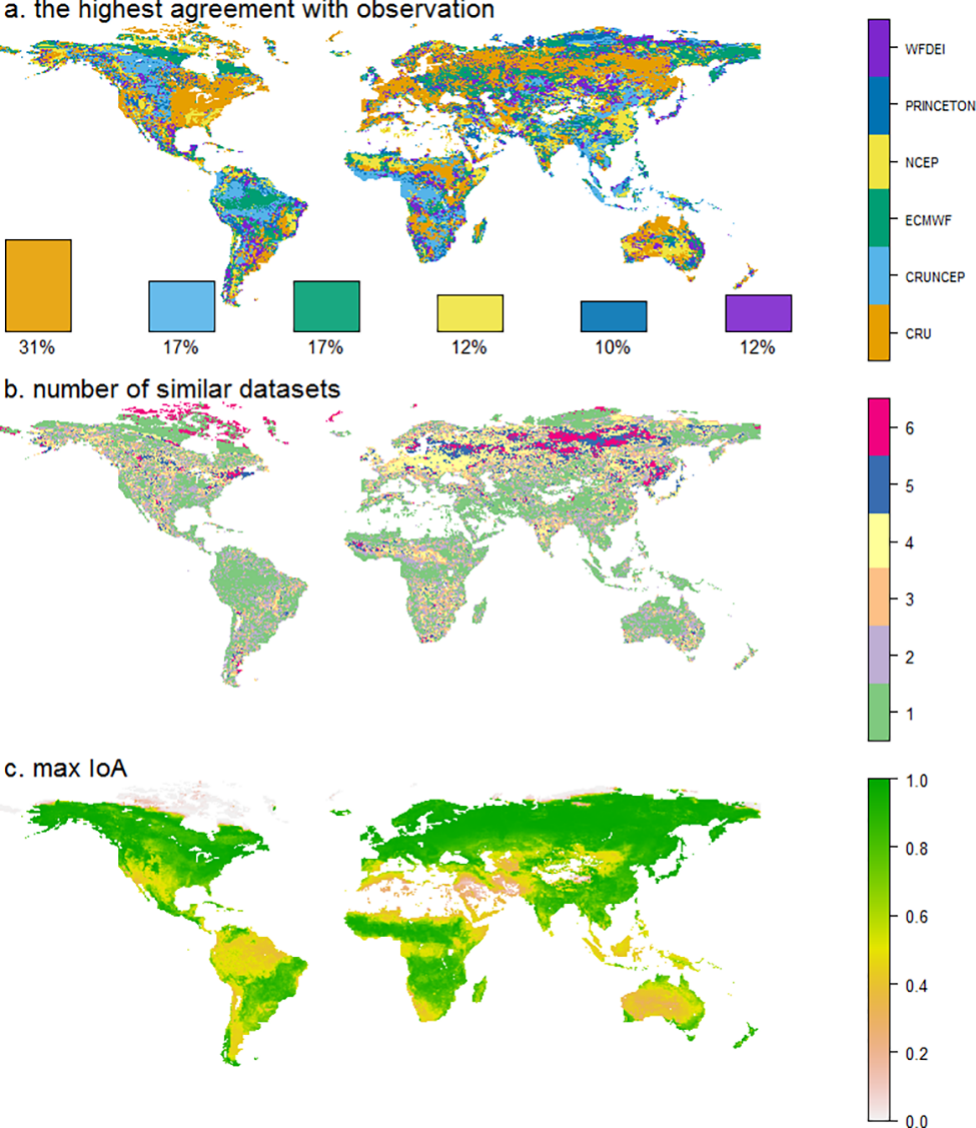
Global maps of climate dataset performance. Panel a) indication of which climate dataset is producing the highest Index of agreement (IoA; calculated at monthly scale) to JUNG11 (1982–2010). The bars show a global total fraction of vegetated grid cells for which the climate dataset is giving the highest IoA. Panel b) shows how many datasets producing GPP simulations with a similar agreement (within 5%) as the one identified in (a). Panel c) displays the maximum IoA between simulated GPP and JUNG11 for each grid cell.

**Fig 2:**
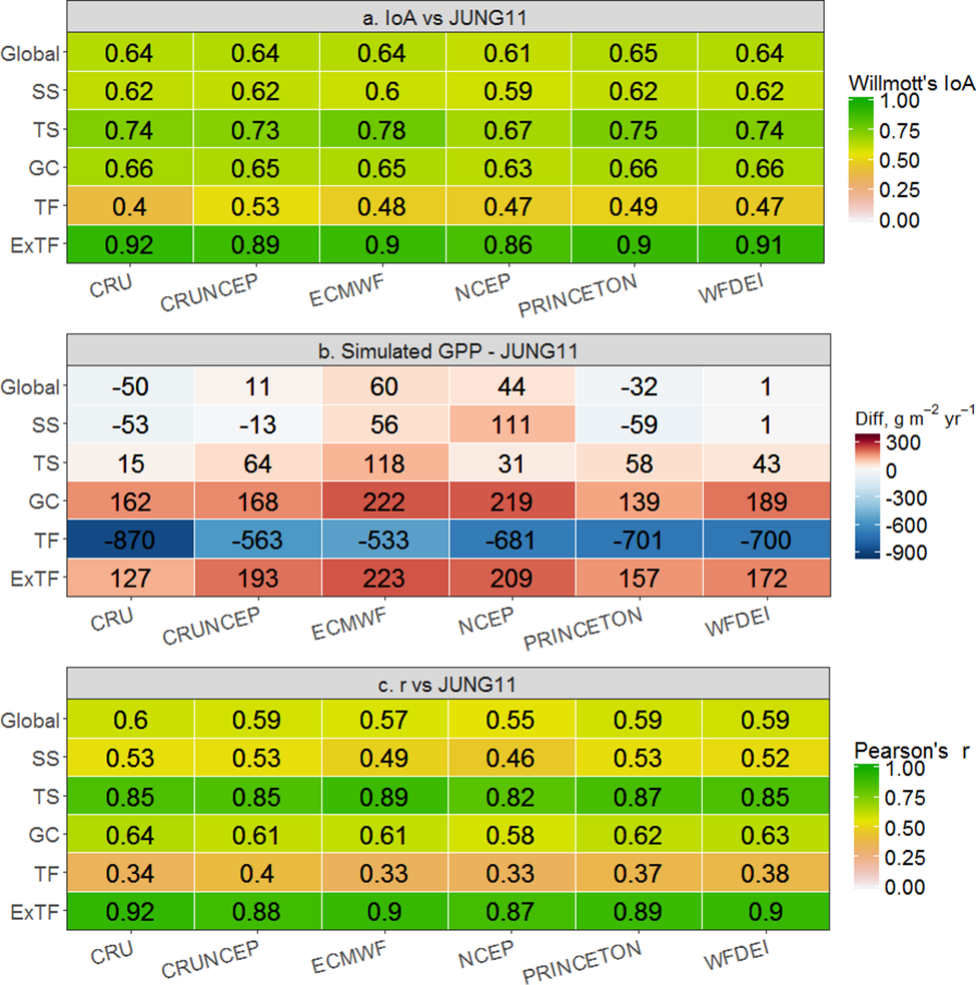
Comparison of monthly IoA, annual mean GPP and monthly temporal correlation during 1982–2010 as estimated by LPJ-GUESS forced by six climate datasets versus the observation-based GPP product JUNG11. Panel (a) shows the IoA, panel (b) shows the average difference and the last panel (c) shows the temporal correlation coefficient between simulated GPP and observations for each land cover class: global; semi-arid ecosystems (SS); tundra and arctic shrub land (TS); grasslands and land under agriculture (GC); tropical forest (TF); extra-tropical forest (ExTF) includes boreal and temperate. The map of land cover classes can be seen in S1 Fig. The spatial distribution of GPP magnitude can be found in S2 Fig.

The agreement between the simulated GPP and the GPP provided by JUNG11 is compared for each grid cell at a 0.5 degree scale using IoA (Fig 1). Our result shows that simulations of GPP using CRU climate data (CRU GPP) have the highest spatial agreement with the reference dataset (31% of global vegetated grid cells where CRU GPP produce the highest IoA). Areas of best agreement are mainly located in the Northern boreal forest but large clusters are also observed in parts of Europe and the United States (Fig 1A). We further found that for a majority of the area (40%, mainly occupied by tropical and dry area), one single dataset was identified with an agreement at least 5% higher (as measured by IoA) than the other datasets (marked as green in Fig 1B). We also found a low IoA for the tropical forest region (Fig 1C), namely tropical Asia, central Africa and tropical South America as well as for desert areas in Africa and Australia. This is in accordance with the simulated GPP showing an expected low mean IoA (<0.5) for Tropical Forests (TF) (Fig 2A) across all datasets with a large bias (Fig 2B) as well as poor temporal correlation (Fig 2C), among which CRUNCEP performs slightly better (r = 0.53). However, at a global scale, the simulated GPP magnitude is relatively close to JUNG11 estimates (Fig 2B). On average simulated GPP is only 6 g m^-2^ yr^-1^ higher than JUNG11, indicating a compensation of regional discrepancies according to overestimation in non-TF and underestimation in TF.

**Fig 3:**
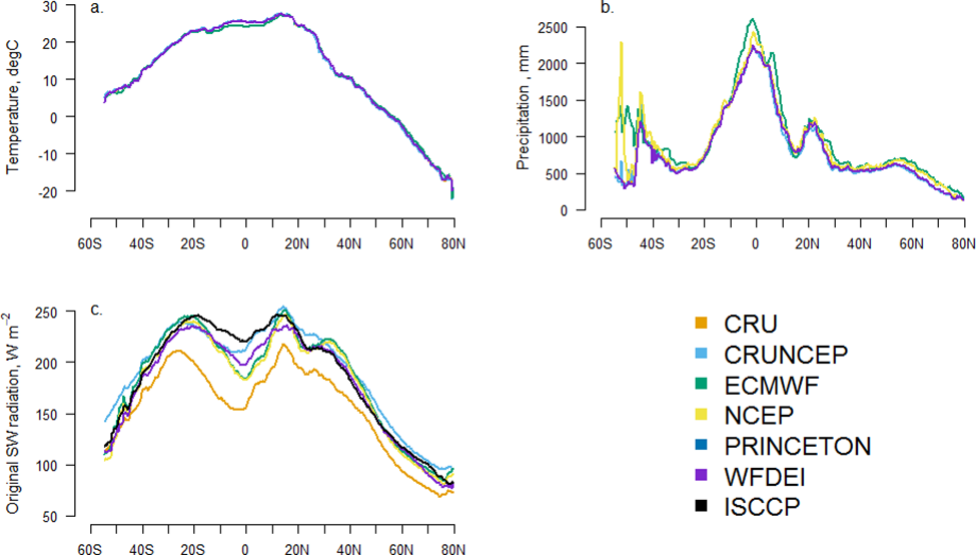
Comparisons of the climatological zonal mean of annual average (1982–2010) of three climate variables among the six climate datasets, i.e. CRU [14], CRUNCEP [30], ECMWF [31], NCEP [32], PRINCETON [17] and WFDEI [18]. The variables are temperature (panel a), precipitation (panel b) and shortwave radiation (panel c). The black line in panel (c) shows the zonal mean of annual average (1984–2000) of ISCCP radiation [33]. The comparisons are conducted for terrestrial areas only. The spatial distribution of each climate variable can be found in S4–S6 Figs.

The comparison of climate data inputs (Fig 3) shows that the zonal mean of the annual temperature is similar among the six climate datasets (Fig 3A) and the precipitation datasets also agree relatively well (Fig 3B) except around the equator and in latitudes below 30-degree South which can be partly attributed to the small number of grid cells in that region. The highest variability is found for the shortwave radiation data (Fig 3C) where the largest discrepancies are found in low latitudes (20°S-20°N), with the CRU shortwave radiation standing out with an average ~14% lower value compared to the mean of the other datasets.

**Fig 4:**
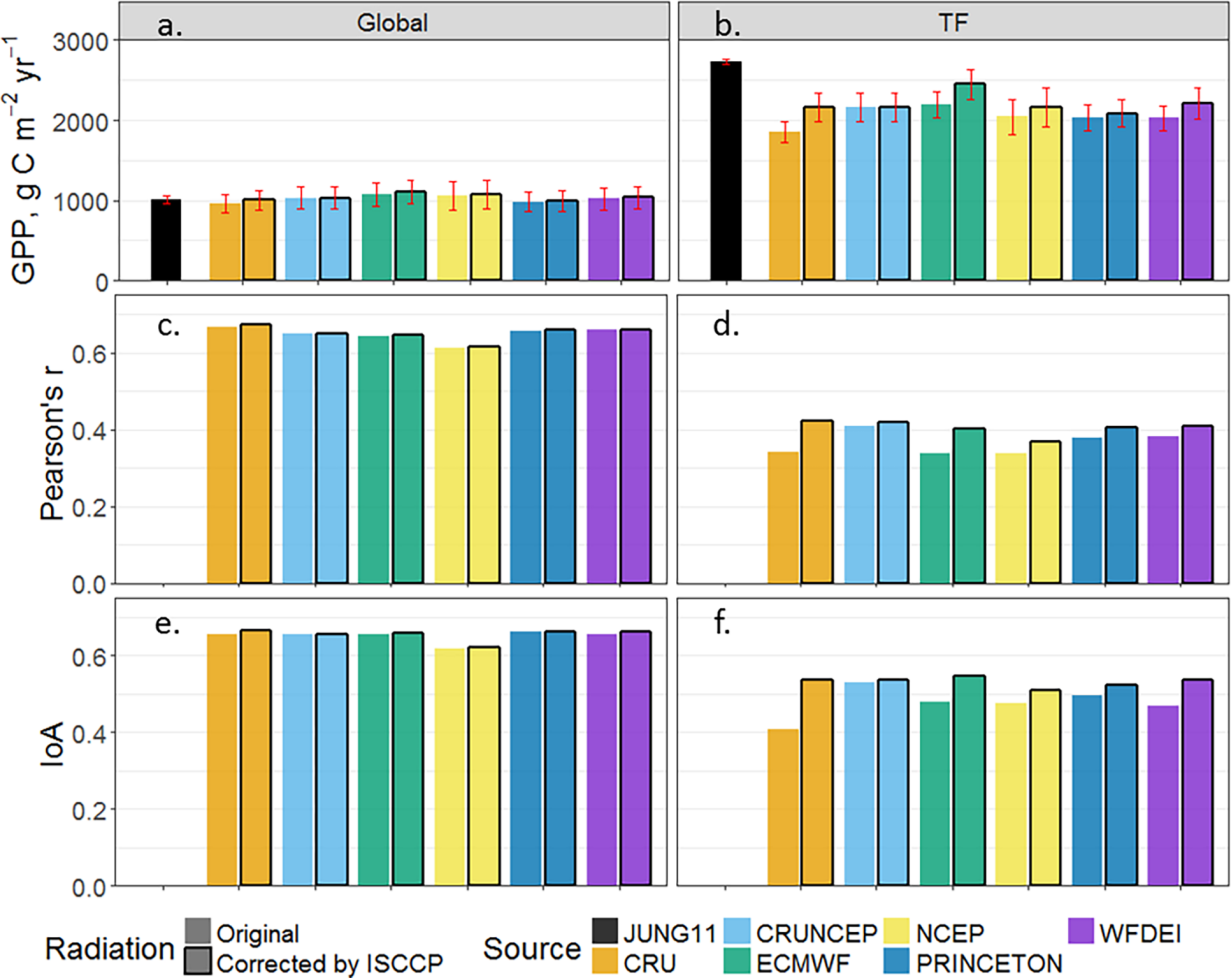
Comparison of annual mean GPP, monthly temporal correlation and monthly IoA during 1982–2010 estimated by LPJ-GUESS before and after tropical forest radiation correction. Panels (a-b) show annual mean GPP, panels (c-d) show temporal correlation and panels (e-f) show the IoA. Bars with a black outline represent simulations based on shortwave radiation corrected by ISCCP data. The extent of tropical forest (TF) is shown in S1 Fig. The red error bars show the inter-annual variability.

To evaluate the influence of the shortwave radiation on the simulated GPP for tropical forest (TF), we bias corrected the shortwave radiation datasets using ISCCP data [33]. The corrected shortwave radiation in tropical forests increased for all climate datasets as compared to the original shortwave radiation. The average increase is lowest for CRUNCEP [30] with 4.9 W m^-2^ and highest for CRU [14] with 57.4 W m^-2^ (S3E Fig). By using the bias corrected shortwave radiation (bar with black outline, Fig 4) over the tropical forest, the average annual GPP shows a 7.5% overall increment while CRU increases with 16.8% and ECMWF shows an increase of 11.7%, and GPP is on average 23.0% closer to the JUNG11 value for all datasets (Fig 4B). This increase in agreement is especially pronounced for the simulation using ECMWF climate data [31], being 48.1% closer to the JUNG11 value after bias correction. There is no significant difference (p>0.05, using one-way ANOVA test) between CRUNCEP simulations with and without bias correction, further confirming that CRUNCEP shortwave radiation has the smallest deviation from the ISCCP data in tropical forest, which could partly explain why CRUNCEP has the relatively higher agreement with JUNG11 over tropical forest in Fig 2A.

The overall effect of correcting shortwave radiation over the tropical forest on simulated global annual mean GPP (Fig 4A) is only 1.2% compared to simulations based on uncorrected shortwave radiation data. The effect of the radiation correction on the temporal correlation (0.6%) and IoA (0.7%) is also negligible on the global scale (Fig 4). The climate induced spread of simulated GPP among climate datasets tested at global scale was reduced from 11.0% to 10.8% by correcting shortwave radiation over the tropical forest.

## Discussion

This study evaluates six climate datasets and their influence on gross primary productivity (GPP) simulated by a biogeochemical model (LPJ-GUESS). Given that GPP is the main driver for a number of vegetation based processes our results can also help to improve the estimation of a variety of other state variables (e.g. net primary productivity). LPJ-GUESS is a well-established biogeochemical model that has been evaluated and applied in a wide range of studies and shows relatively similar behavior and predictive skills compared to other biogeochemical models [43–45]. This is especially true for GPP, given that most biogeochemical models (e.g. HYLAND, LPJ-DGVM, OCN, ORCHIDEE, SDGVM and TRIFFID) use the same photosynthesis model [46] at their core [9, 39, 47]. LPJ-GUESS may thus be considered a generic representative for biogeochemical models as a group and very likely reproduces spatial and temporal characteristics of primary productivity.

The datasets investigated are all widely used in studies focusing on modeling the carbon cycle and the results show that the differences are most pronounced for shortwave radiation. CRU shortwave radiation is calculated from cloud cover, which is derived from observations of sun hours, by using the method of Harris *et al*. [29]. CRUNCEP shortwave radiation on the other hand is rescaled from the NCEP-NCAR [48] reanalysis data by using the MTCLIM model [49], which reduces the magnitude of NCEP-NCAR shortwave radiation to better match observed radiation at FLUXNET sites [30]. The reanalysis shortwave radiation from ECMWF and NCEP (here we use DOE II which differs from NCEP-NCAR) are produced by different radiative transfer schemes from Mlawer *et al*. [50] and Chou [51], respectively. These schemes describe how solar irradiance is attenuated by the absorption and scattering (due to e.g. water vapor, oxygen, trace gases, clouds, and aerosols) when passing through the atmosphere before reaching the land surface. PRINCETON shortwave radiation is based on interpolating the NCEP-NCAR reanalysis product and downscaling to 0.5 degree prior to bias correction using CRU data [17]. WFDEI shortwave radiation is derived from the ERA-40 reanalysis product [52] and the dataset is adjusted by using CRU cloud cover [53]. Given the manifold methodological differences, it is a challenge to determine whether all datasets are equally reliable or if any of them is better suited for a certain study region or purpose than others.

Overall, CRU driven GPP results in the best agreement with JUNG11 for the largest area compared to the other climate datasets, which may be due to the fact that the JUNG11 has been generated by incorporating CRU data to some extent [34]. However, still in almost 70% of the vegetated area JUNG11 agrees better with one of the other climate datasets used as input for simulating GPP. We also used the observation-based MODIS GPP product [54] in our analyses. Even that the MODIS GPP algorithm includes the NCEP dataset (one of tested datasets) as an input of daily meteorological data, the results agreed that CRU is a better climate forcing in more grid cells than the other datasets tested (S7 Fig). Considering that the long-term observation and climatic information used in JUNG11 is not entirely from CRU, we decided to use JUNG11 as the benchmark of this study. Our study shows that the specific choice of the climate dataset to be used for driving the biogeochemical model (out of the six historical datasets investigated) is associated with smaller spread in simulated GPP at the global scale than the spread at the regional scale (Fig 2), which indicates that the choice of the climate dataset for estimating global GPP is less critical as when estimating GPP at the regional scale. The largest disagreement of GPP between LPJ-GUESS simulations and JUNG11, is found in the tropical region. This pattern is consistent with findings that the tropical region has the largest differences in GPP estimates between process-based models and data-driven methods [55–57]. We also found the largest disagreement between simulated GPP in the tropical region, which is attributed to the large bias of shortwave radiation among investigated climate datasets and the high sensitivity of GPP to shortwave radiation over the tropics [12]. Wu *et al*. [12] also showed that differences in shortwave radiation caused large differences in simulated GPP over tropical regions when using LPJ-GUESS, which is likely to be similar for other biogeochemical models [10]. The bias of shortwave radiation in tropical areas has been attributed to the sparse meteorological station network [58] and to the high uncertainties in radiation transfer, cloud cover and cloud morphology when producing the climate datasets [11, 59].

We also show that a bias correction of shortwave radiation data (using the ISCCP radiation data) in the climate datasets causes the simulated GPP to markedly increase in the tropical region (e.g. CRU and ECMWF simulations), reducing the gap in simulated GPP compared to JUNG11. This again suggests that shortwave radiation products currently available for tropical regions remain highly uncertain. In order to accurately simulate GPP in tropical regions (which are known to be primarily constrained by incoming solar radiation) we suggest improving shortwave radiation of the tested datasets, e.g. by bias correcting with advanced radiation data from ISCCP. ISCCP reduced cloud effects on radiation by using an advanced radiative transfer model [33, 40], which makes ISCCP radiation data more reliable in cloud-prone tropical forest areas than the radiation from the tested climate datasets (except CRUNCEP). Following the bias correction, we found no significant change for the CRUNCEP simulation, which suggested that the shortwave radiation from CRUNCEP has equally high quality as ISCCP. The high quality of the shortwave radiation data from CRUNCEP in the tropic is likely to be one of the reasons for more grid cells of highest IoA being derived from the CRUNCEP stimulation in Fig 1A. Furthermore, we found that correcting only for shortwave radiation is not enough to produce an exact match with observation-based estimates as there is still a substantial gap between model simulations and JUNG11. The ECMWF dataset, characterized by the highest precipitation, also showed the highest agreement with JUNG11 after shortwave radiation correction, which implies that not only the radiation but also the precipitation over tropical areas might be underestimated in the climate datasets tested. Previous studies [10, 12] also found that GPP was sensitive to precipitation in tropical areas. Therefore, if aiming at producing a set of climate variables to minimize the discrepancy between modelled and observed GPP, we recommend also to improve the precipitation variable of tested climate datasets (e.g. CRUNCEP which has high quality radiation and temperature data) e.g. by bias correction using high quality precipitation data (e.g. TRMM [60]) in tropical region.

Although correcting the shortwave radiation over the tropical forest reduced the climate induced spread of simulated GPP among climate datasets tested at global scale from 11.0% to 10.8%, which is within the range of 9%-20% [10–12], the aim of the bias correction was not to narrow the climate induced spread. We would expect that if correcting all of the three climate variables there will be no climate induced spread among climate datasets tested. Bias correction is one way that could help improving the climate variable of a climate dataset in a certain study area, by using ISCCP, TRMM or other available high quality data. However, the correction of a given climate variable within a climate dataset should be done with caution, as improving a single variable from a climate dataset may introduce an imbalance in relation to other co-varying climate variables of that dataset. Therefore, we consider it preferably to first select a suitable climate dataset for a study area and then, if deemed necessary, a given variable of this dataset can additionally be bias-corrected.

In order to avoid over-interpretation of model-data mismatches, it is mandatory to also consider the limitations of the reference data. JUNG11 GPP used in this study was assumed to represent the “true” GPP but inevitably also includes systematic and random errors and uncertainties. For instance, uncertainties of flux measurements derived from discriminating low and well mixed fluxes [61], estimation of missing values [62], and flux partitioning (e.g. partition the observed net ecosystem exchange (NEE) in to GPP and ecosystem respiration) [63, 64]. These uncertainties, furthermore, propagate when extrapolating to the globe by the MTE approach [34]. One additional complication arises from the possibility that JUNG11 also performs poorly over tropical areas and that disentangling uncertainties within the GPP simulated by LPJ-GUESS and JUNG11 might be impossible.

One additional limitation of this study is related to the evaluation method. IoA is used as the main metric for the evaluation since it combines patterns like the Pearson correlation coefficient and information on the magnitude of deviations. However, it is known to be sensitive to extreme values due to the squared differences which potentially over-weighs the influence of the differences between model prediction and observation [65]. Hence, we have complemented this statistical measure with calculations of the average difference and correlation coefficient.

## Conclusion

This study evaluates the performance of the six most commonly used climate datasets (CRU, CRUNCEP, ECMWF, NCEP, PRINCETON, WFDEI) in estimating terrestrial GPP within a spatially explicit biogeochemical model by using independent observation-based GPP data. Our study highlights the need to improve the incoming shortwave radiation estimates from most of the climate datasets tested (except CRUNCEP) in tropical areas in order to improve GPP estimates over tropical regions. Our results also allow the assessment of the suitability of climate datasets with respect to a given research purpose and study area, e.g. the CRUNCEP dataset works better in tropical regions for simulating GPP (values being in agreement with observation-based GPP), while the choice of the climate dataset for simulating GPP in Europe is less critical.

## Supporting information

S1 Fig Map of land cover classes. The source of the data derived from Ahlström et al. [39] and Wu et al. [12]. The percentage values at the bottom of the map show the fraction of each land cover class in relation to the global terrestrial area (excluding Greenland).
(TIF)

S2 Fig Comparison of annual mean GPP during 1982–2010 from model simulations by using different climate datasets and observation-based estimate (JUNG11). a. global GPP linear trends. b. GPP zonal means. c-h maps of spatial difference of annual mean GPP between simulations forced with different climate datasets and observations (g C /m^-2^).
(TIF)

S3 Fig Annual mean shortwave radiation during 1982–2010 globally and stratified by land cover classes. Bars with a black outline represent the simulations based on shortwave radiation is corrected by ISCCP data.
(TIF)

S4 Fig Comparison of annual temperature from the climate datasets tested. a. global annual trends, b. zonal means, c-j. spatial distribution of mean annual temperature.
(TIF)

S5 Fig Comparison of annual total precipitation from the climate datasets tested. a. global annual trends, b. zonal means, c-j. spatial distribution of mean annual precipitation.
(TIF)

S6 Fig Comparison of annual shortwave radiation from the climate datasets tested. a. global annual trends, b. zonal means, c-j. spatial distribution of mean annual shortwave radiation.
(TIF)

S7 Fig Global maps of climate dataset performance. Panel a, c, and e show the results when using MODIS GPP (2000–2010) as the benchmark, and panel b, d and f show the results when using JUNG11 GPP (1982–2010) as the benchmark. For the description of the figure is referred to Fig 1 in the main text.
(TIF)
