# Supplementary figures and images for: Effect of climate dataset selection on simulations of terrestrial GPP: Highest uncertainty for tropical regions
Source: PLoS One. 2018 Jun 21;13(6):e0199383. doi: 10.1371/journal.pone.0199383 (PMC6013155; doi:10.1371/journal.pone.0199383)

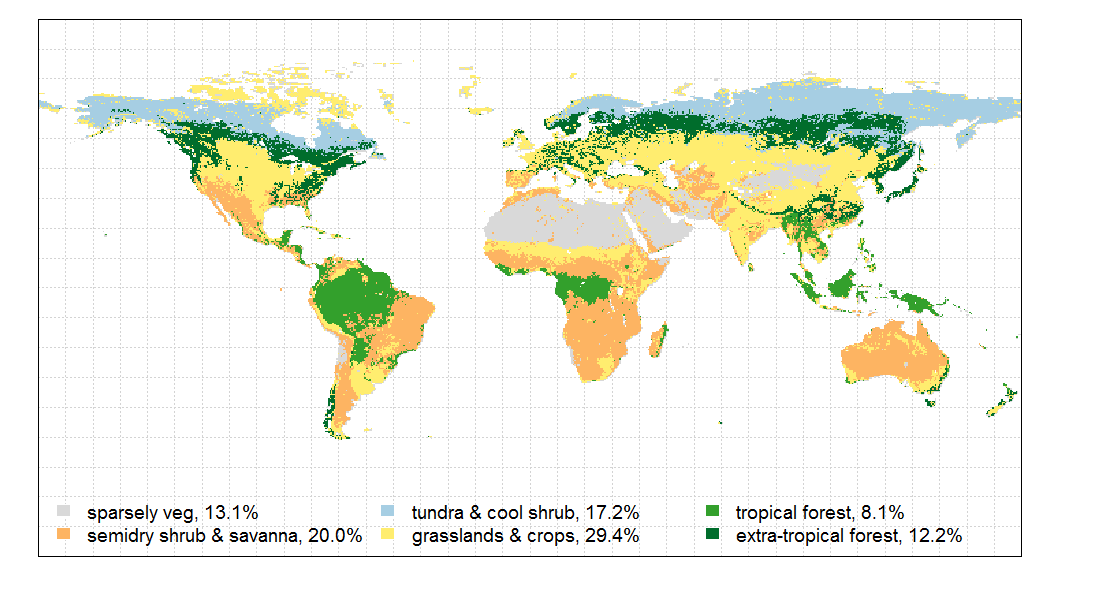

Supplement: S1 Fig — The source of the data derived from Ahlström et al. [39] and Wu et al. [12]. The percentage values at the bottom of the map show the fraction of each land cover class in relation to the global terrestrial area (excluding Greenland). (TIF) [file pone.0199383.s001.tif]

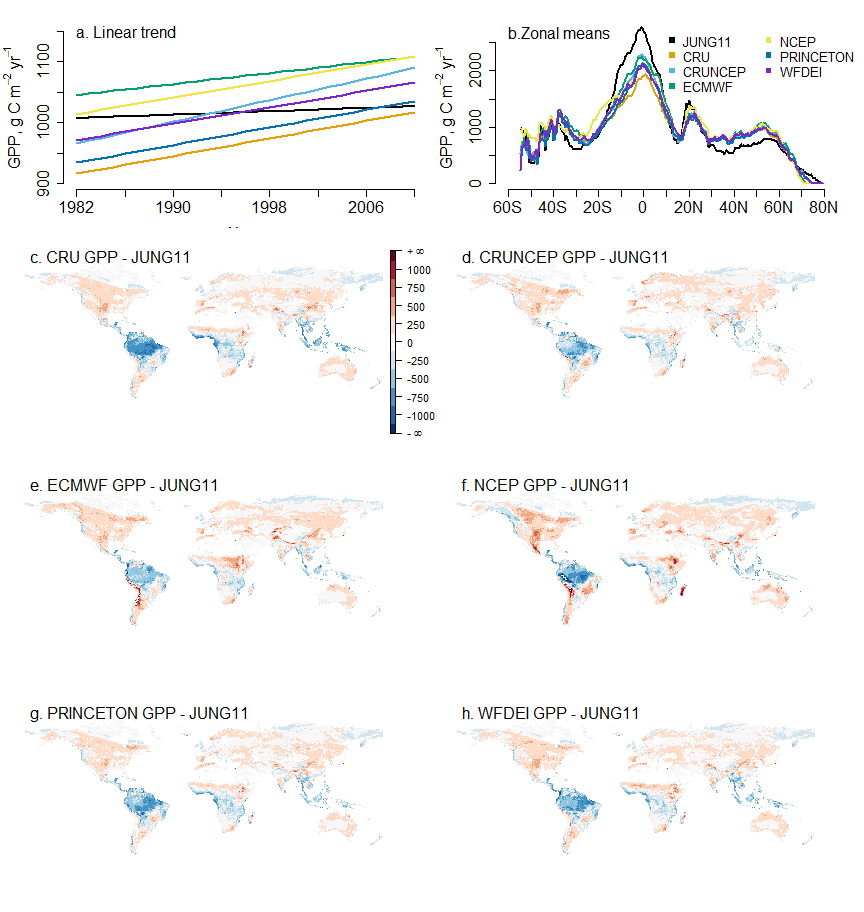

Supplement: S2 Fig — a. global GPP linear trends. b. GPP zonal means. c-h maps of spatial difference of annual mean GPP between simulations forced with different climate datasets and observations (g C /m-2). (TIF) [file pone.0199383.s002.tif]

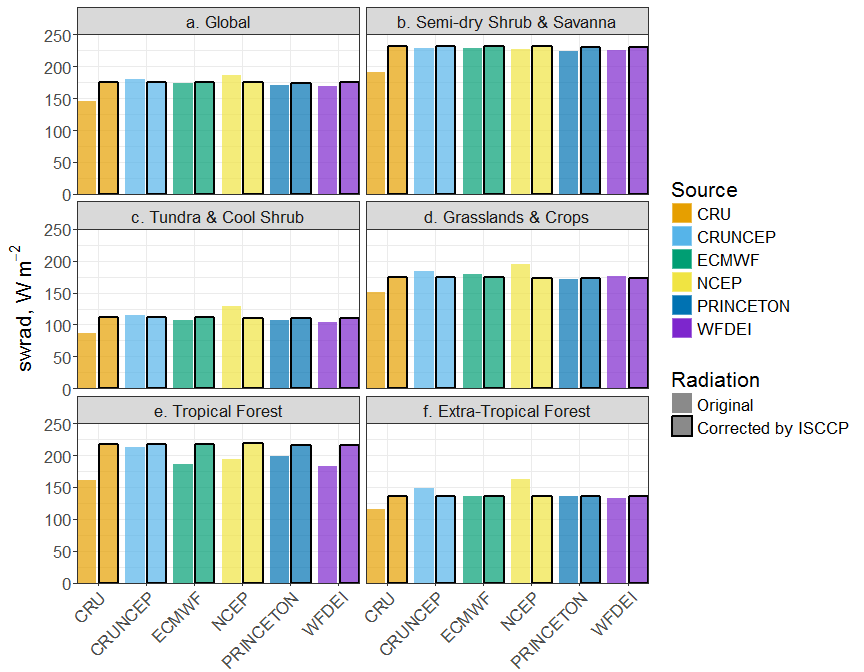

Supplement: S3 Fig — Bars with a black outline represent the simulations based on shortwave radiation is corrected by ISCCP data. (TIF) [file pone.0199383.s003.tif]

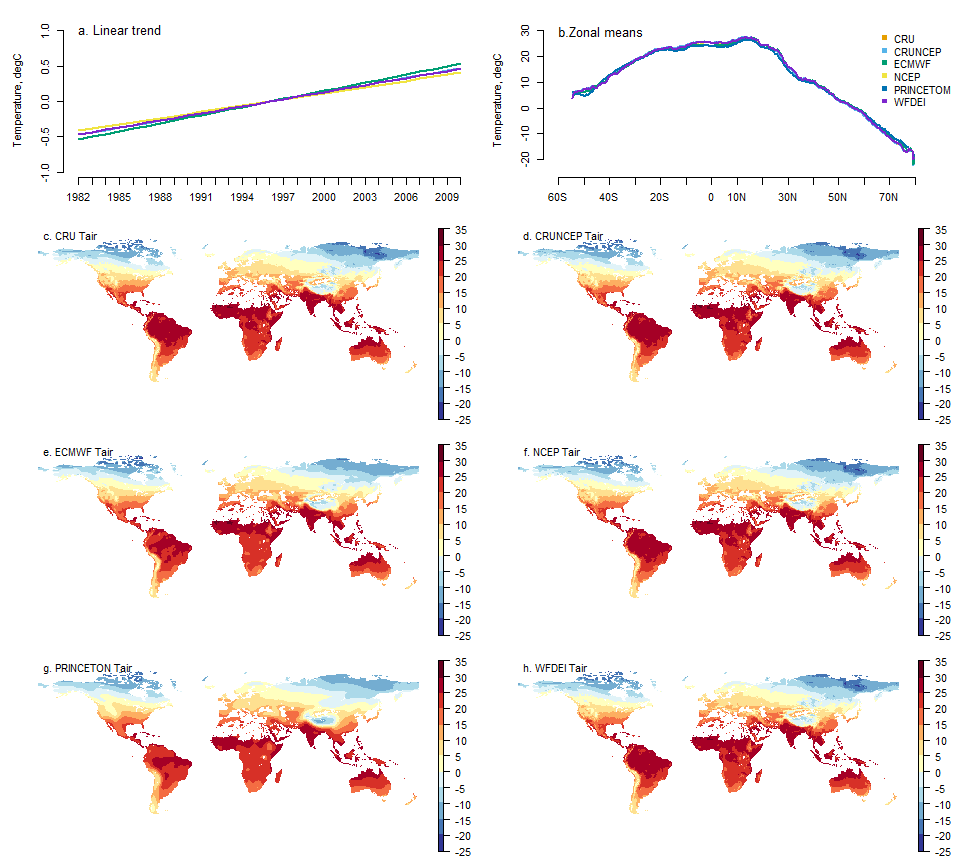

Supplement: S4 Fig — a. global annual trends, b. zonal means, c-j. spatial distribution of mean annual temperature. (TIF) [file pone.0199383.s004.tif]

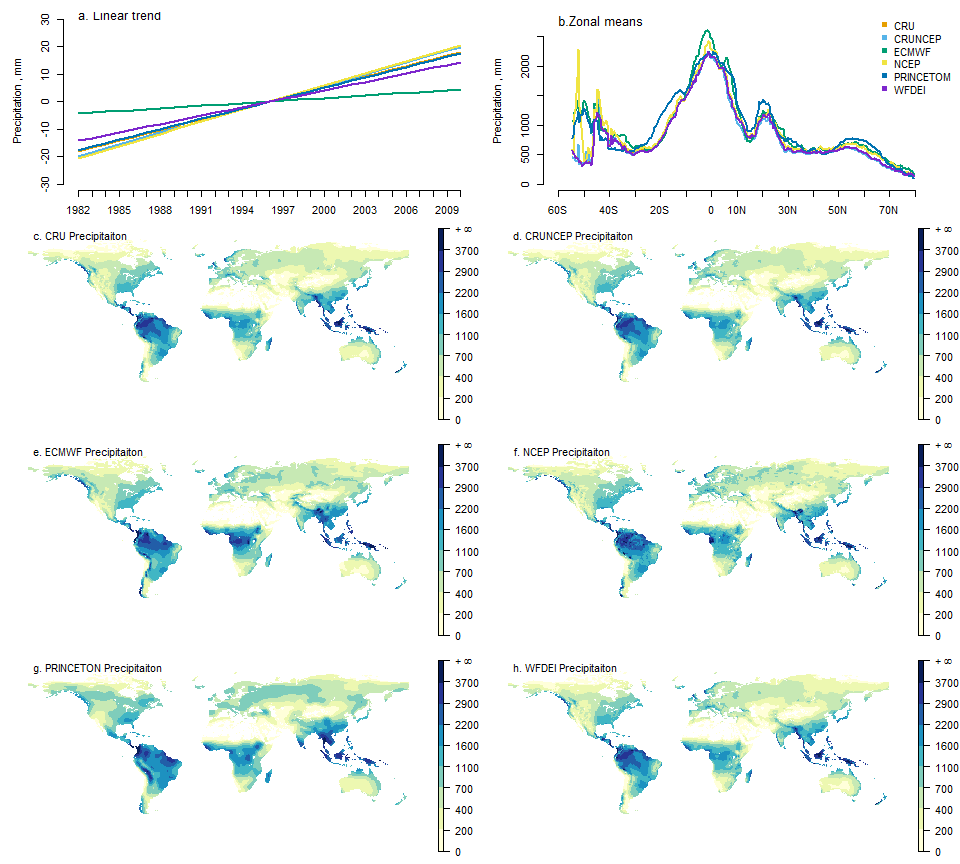

Supplement: S5 Fig — a. global annual trends, b. zonal means, c-j. spatial distribution of mean annual precipitation. (TIF) [file pone.0199383.s005.tif]

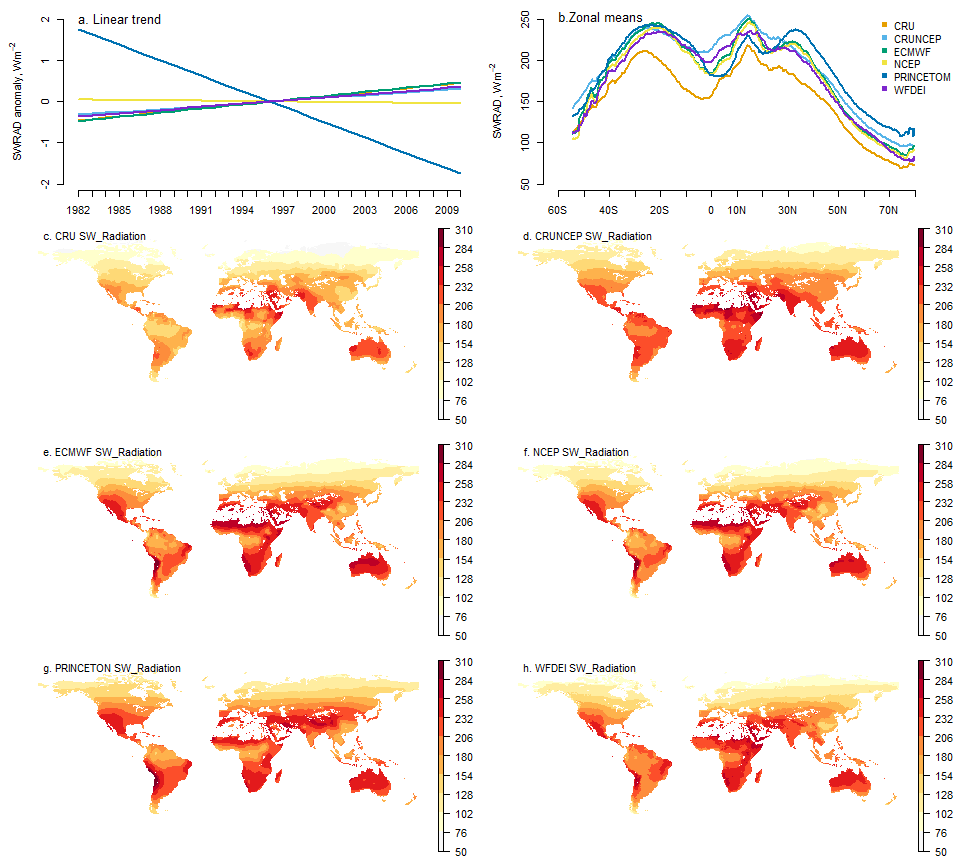

Supplement: S6 Fig — a. global annual trends, b. zonal means, c-j. spatial distribution of mean annual shortwave radiation. (TIF) [file pone.0199383.s006.tif]

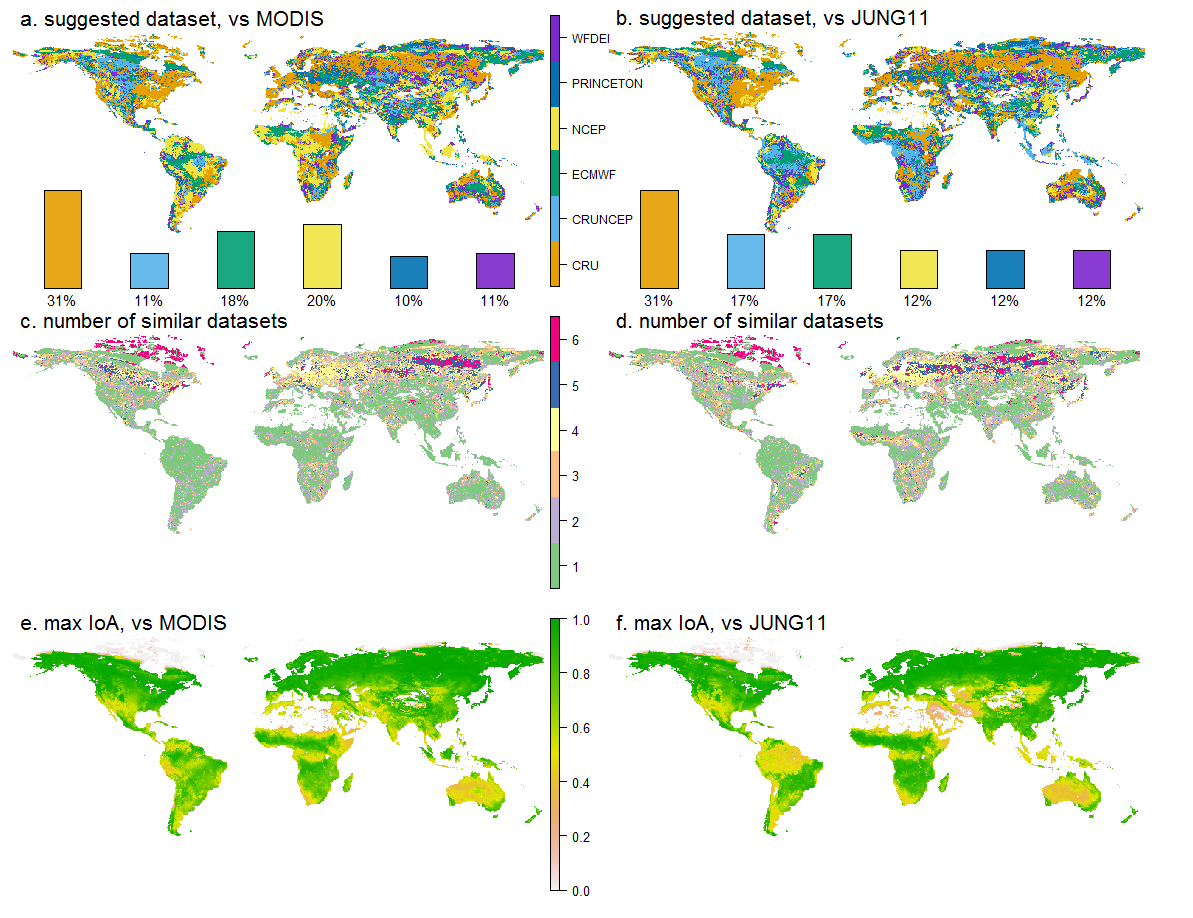

Supplement: S7 Fig — Panel a, c, and e show the results when using MODIS GPP (2000–2010) as the benchmark, and panel b, d and f show the results when using JUNG11 GPP (1982–2010) as the benchmark. For the description of the figure is referred to Fig 1 in the main text. (TIF) [file pone.0199383.s007.tif]
